# The Risk of Bleeding in Small/Straight Esophageal Varices with Red Color Sign on Endoscopy: A Retrospective Analysis from the Natural Course

**DOI:** 10.3390/healthcare10071193

**Published:** 2022-06-26

**Authors:** Kazunori Nagashima, Atsushi Irisawa, Ken Kashima, Fumi Sakuma, Takahito Minaguchi, Akira Yamamiya, Akane Yamabe, Koki Hoshi, Keiichi Tominaga, Makoto Iijima, Kenichi Goda

**Affiliations:** Department of Gastroenterology, Dokkyo Medical University School of Medcine, 880 Kitakobayashi, Mibu, Tochigi 321-0293, Japan; n-kazu@dokkyomed.ac.jp (K.N.); irisawa@dokkyomed.ac.jp (A.I.); ken-k@dokkyomed.ac.jp (K.K.); sakuma-f@dokkyomed.ac.jp (F.S.); takahito@dokkyomed.ac.jp (T.M.); akira-y@dokkyomed.ac.jp (A.Y.); yamabe@dokkyomed.ac.jp (A.Y.); hoshi@dokkyomed.ac.jp (K.H.); mkiijima@dokkyomed.ac.jp (M.I.); goda@dokkyomed.ac.jp (K.G.)

**Keywords:** endoscopic injection sclerotherapy, endoscopic variceal ligation, esophageal varices, red color sign, small straight varices

## Abstract

Red color sign-positive (RC-positive) esophageal varices present a high bleeding risk, necessitating prophylactic treatment. Among RC-positive esophageal varices, those classified morphologically as small straight varices (Form level 1: F1) are difficult to treat. Moreover, the appropriate time for therapeutic intervention remains undefined. This study assessed the bleeding risk in RC-positive F1 esophageal varices. After extracting 541 cases of F1 esophageal varices diagnosed during 1 January 2012–29 February 2020, 76 cases of RC-positive F1 esophageal varices were divided into two groups in terms of treatment intervention at diagnosis: 49 cases with (treatment group) and 27 cases without (follow-up group). We assessed the bleeding rates, bleeding-associated factors, and early-bleeding-associated factors. The treatment group’s bleeding rate was 10% (5/49). The follow-up group’s bleeding rate was 78% (21/24). The subsequent bleeding rate was low in the treatment group (*p* < 0.001). The median period of sustained absence of bleeding was longer in the treatment group than in the follow-up group (1156 [274–1582] days vs. 105 [1–336] days; *p* < 0.001). In the follow-up group, a significant number of bleedings had varices that included a hematocystic spot (HCS) as RC or combined with RC (*p* = 0.017). Early bleeding occurred often in varices that included HCS or combined with RC (*p* = 0.024). Red wale marking (RWM) only was not a factor of early bleeding (*p* = 0.012). In conclusion, RC-positive varices should be treated even as F1 varices. Patients with RWM only show the possibility of not accepting early treatment intervention. A fast response is crucially important in HCS cases because of its associated bleeding and early bleeding.

## 1. Introduction

Portal hypertension is accompanied by various symptoms, including esophagogastric varices, ectopic varices, ascites, hypersplenism, hepatic encephalopathy, and portal vein thrombosis. Esophageal varices are a pathological condition caused by the development of collateral circulation in the esophagus as a result of portal hypertension. Bleeding of esophageal varices can be fatal. For that reason, any information supporting bleeding risk prediction is extremely useful. Factors involved in esophageal variceal bleeding reportedly include alcohol consumption, hepatic reserve, pharmacological agents, and endoscopic findings, including reddening.

Because endoscopy can be performed safely, attempts have been undertaken to predict bleeding based on the variceal form, site, and surface properties [1]. Large esophageal varices with a morphological form (F) categorized as enlarged tortuous varices (form level 2: F2) or largest varices (form level 3: F3) according to the classification of the Japan Society for Portal Hypertension [2], as well as with the red color sign (RC) irrespective of the variceal form, are regarded as particularly at risk of bleeding. Prophylactic treatment is recommended in such cases [3,4,5]. However, no clear evidence exists to indicate whether small varices (straight varices, form level 1: F1) with RC are at high risk of bleeding.

The American Association for the Study of Liver Diseases (AASLD) guideline recommends the use of nonselective beta-blockers (NSBBs) for patients with high-risk small esophageal varices (with red wale marks) [6]. In fact, even for varices with an F1 form, treatment aimed at bleeding prevention must be considered if the RC sign is positive, even for short varices. Moreover, if endoscopic intervention is available according to the patient’s status, endoscopic injection sclerotherapy (EIS) and/or endoscopic variceal ligation (EVL) might be considered for reliable bleeding prevention. Nevertheless, the appropriate timing of endoscopic intervention without administration of NSBB remains unclear.

The research question of this study is in ascertaining the risk of bleeding in RC-positive F1 esophageal varices to clarify appropriate timing for endoscopic treatment in such cases. To this end, we retrospectively examined the natural course of F1 and RC-positive esophageal varices without NSBBs.

## 2. Materials and Methods

### 2.1. Patients

**Figure 1:**
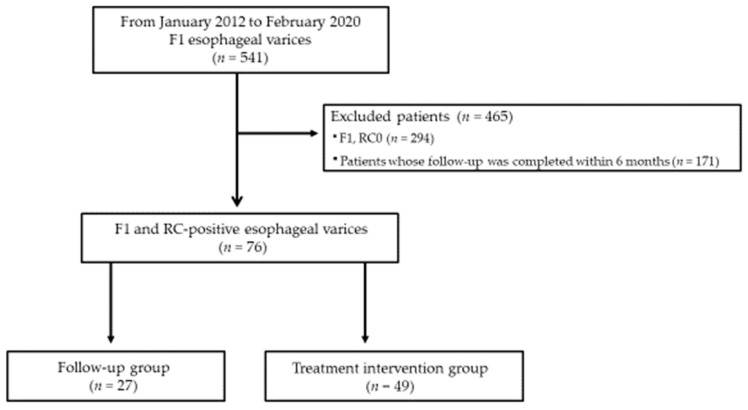
Flowchart showing studied patients.

From past medical records preserved at Dokkyo Medical University Hospital from 1 January 2012 through 29 February 2020, we extracted the data of 541 patients diagnosed with F1 esophageal varices, including those who had completed treatment. Among these patients, RC sign-negative cases (294 cases) were excluded. Among the cases of RC-positive F1 esophageal varices, the following were excluded: cases that showed no morphological recurrence or bleeding or for which follow-up of the clinical course was not completed within 6 months, as well as cases for which CT showed no confirmation of the presence of inflow vessels. Ultimately, 76 cases and their respective treatment histories were examined. None of the related 76 patients had undergone administration of NSBBs. The 76 patients with RC-positive F1 esophageal varices were divided into two groups: 49 patients (treatment intervention group) received treatment interventions consisting of EVL or intravascular injection of ethanolamine oleate (EIS) or EIS with ligation (EISL) at the time of diagnosis; 27 patients (follow-up group), who received no treatment intervention from the time of diagnosis, showed disease progression limited to the natural history of the condition (Figure 1).

The observation period started when esophageal varices were detected and was set to end on 30 September 2020. Observation was terminated if the follow-up using upper gastrointestinal endoscopy showed morphological recurrence or bleeding. The patients were followed up regularly with outpatient care every 1–2 months to confirm the variceal bleeding status. All patients visited the outpatient care after 30 September 2020, and at that time, the presence or absence of bleeding events as of September 30 was confirmed by direct interview. No patients died during their course.

### 2.2. Endoscopic Findings

**Figure 2:**
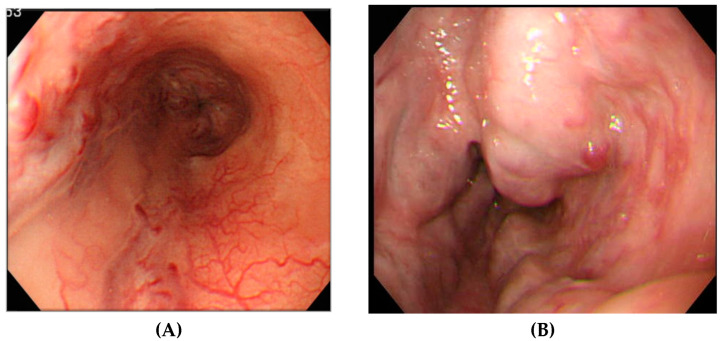
RC findings in esophageal varices. Cherry red spot (CRS) and red wale marking (RWM) (**A**). Two varices were found at 9 o’clock and 6 o’clock, respectively. Several reddened spots, which consisted of CRS, were found at 9 o’clock. Thin linear blood vessels, which consisted of RWM, were found at 6 o’clock. Hematocystic spot (HCS) (**B**). The varices found at 2 o’clock were thick and showed strong development. The thick blood vessel prominence, which is an HCS, is visible on the varices.

Endoscopically, esophageal varix form (F) and the appearance of RC signs were classified according to The General Rules for Study of Portal Hypertension (The Japan Society for Portal Hypertension, Third Edition, 2013) [2]: F1 was defined as straight varices. The RC sign was defined as existing when erythrogenic findings were visible on the varices endoscopically. The RC sign was classified subjectively into three categories of appearance and four categories of severity. The three categories of appearance were cherry red spot (CRS), red wale marking (RWM), and hematocystic spot (HCS) (Figure 2). The four categories of severity were the following: RC0, no erythrogenic finding; RC1, a few lo-calized erythrogenic findings; RC2, between RC1 and RC3; and RC3, many erythrogenic findings through 360°.

### 2.3. Study Design

This is a retrospective study of F1 varices with RC between the treatment intervention group and the follow-up group. The primary endpoints of this study consisted of factors related to bleeding and early bleeding in the follow-up group. Early bleeding was defined as bleeding occurring earlier than the median bleeding time. Secondary endpoints included the bleeding rate of esophageal varices and the time to bleeding (period of sustained absence of bleeding), respectively, in the treatment intervention group and the follow-up group. For this study, patients with bleeding were defined as those whose upper gastrointestinal endoscopy confirmed the bleeding point. Antithrombotic therapy included antiplatelet agents: low-dose aspirin (LDA), antiplatelet agents such as thienopyridines or cilostazol, or anticoagulants, such as warfarin, and direct oral anticoagulants (DOACs). Patients who took oral proton pump inhibitors (PPIs) as acid suppressants before bleeding were regarded as oral PPI-treated patients, irrespective of the type of PPI they took. Regarding alcohol consumption, past alcohol consumption history was excluded. Patients who continually consumed an average of 40 g alcohol/day were defined as alcohol-consuming patients. Variceal inflow vessels and portosystemic shunts (e.g., gastrorenal shunts and splenorenal shunts) were assessed using plain or contrast-enhanced computed tomography. The assessment of shunts was based only on their presence or absence. Variceal inflow vessels were categorized as three types: left gastric vein, posterior gastric vein, and the presence of multiple vessels of the above types. Treatment history was based on whether the patient received EVL alone or endoscopic injection sclerotherapy ligation (EISL), combining the use of sclerosants (ethanolamine oleate, EO) and endoscopic variceal ligation (EVL) [7].

This study, which was approved by the Institutional Review Board of Dokkyo Medical University Hospital (approval number R-36-6J), was conducted in accordance with the principles of the Declaration of Helsinki and registered on the University Hospital Medical Network Clinical Trials Registry [UMIN R000052875]. Because this was a retrospective study, informed consent was not based on written consent, but through web publication of information related to the execution of this study, in compliance with the “ethical guidelines for medical research conducted on human subjects.” Additionally, we specified that whenever a patient did not agree with the usage of their data, suspension of the use of their data was available upon submission of an application.

### 2.4. Statistical Analysis

Statistical processing was performed using statistical software (IBM SPSS Statistics 21^®^; IBM Japan Ltd., Armonk, NY, USA). Continuous variables, such as age, were tested using the Mann–Whitney U test. Recurrences were compared using the x-square test (including Cramer’s V). For the x-square test, *p*-values < 0.05 were inferred as significant. Data were regarded as reliable when Cramer’s V was ≥ 0.3 (especially when it was closer to 0.5).

## 3. Results

### 3.1. Patient Characteristics

The overall patient characteristics are shown in Table 1. The characteristics of patients in the treatment intervention group and follow-up group are presented in Table 2.

The overall median age of patients was 62 (43–82) years. The numbers of male and female patients were, respectively, 49 and 27. Regarding comorbidities, hypertension was found in 17 patients (22%), diabetes mellitus was found in 21 patients (28%), and hyperlipidemia was found in four patients (6%). Atrophic gastritis was found in 53 patients (70%). Only four patients with hyperlipidemia were taking statins. Patients received antithrombotic therapy in 13 cases (17%). There were 10 patients treated with oral NSAIDs (13%). There were 61 patients treated with oral PPIs (82%). Continuous alcohol consumption was reported in 10 cases (13%). Hepatitis C virus (HCV) was the most common causative disease of liver cirrhosis, with 18 cases (24%) reported in this study.

Portal vein thrombosis was reported for four cases (5%). Hepatocellular carcinoma was shown for 18 cases (24%). There were nine (12%) patients with portal vein invasion (VP). Details related to VP show that VP1, 2, 3, and 4 respectively accounted for three cases (4%), four cases (6%), one case (1%), and one case (1%). Collateral circulation, such as gastrorenal shunt, was found in 32 cases (42%). Child–Pugh classification A was noted for 36 cases (47%). The mean platelet count was 11.5 (×10^4^/µL ± SD:5.4). The most frequently involved vessel for variceal inflow was the left gastric vein, with 57 cases (75%). Patients with a history of treatment for varicose veins accounted for 17 cases (63%). The RC findings are shown in Table 2. No difference was found in the backgrounds of the treatment intervention group and the follow-up group.

#### Bleeding Rate and Period of Sustained Absence of Bleeding

As Table 2 shows, no difference was found between the backgrounds of the intervention group and the follow-up group. Among the cases of F1 varices with RC-positive, the bleeding rate in the treatment intervention group was 10% (5/49). The bleeding rate in the follow-up group was 78% (21/27); the subsequent bleeding rate was lower in the treatment intervention group (*p* < 0.001). Among bleeding cases, the median period of sustained absence of bleeding was 1156 (274–1582) days in the treatment intervention group and 105 (1–336) days in the follow-up group; the period of sustained absence of bleeding was longer in the treatment intervention group (*p* < 0.001).

### 3.2. Factors Associated with Bleeding and Early Bleeding in the Follow-up Group

#### 3.2.1. Bleeding-Associated Factors

The follow-up group cases were divided into 21 bleeding cases and 6 non-bleeding cases. The factors associated with bleeding in the natural course of esophageal varices (age, sex, comorbidities, receiving or not receiving antithrombotic therapy, alcohol consumption, hepatic functional reserve, VP, treatment history, shunt, platelet count, differences in variceal inflow vessels, background liver, and endoscopic findings) were examined (Tables S1–S3).

No significant difference was found between the bleeding and non-bleeding cases in terms of patient characteristics, hepatic functional reserve, and background liver. Endoscopic findings showed no significant differences between the bleeding and non-bleeding cases in terms of RC, but those with treatment history tended to be more common in the non-bleeding group (*p* = 0.057). Our findings showed less CRS and less RWM among the bleeding cases (*p* = 0.04 and *p* = 0.50, respectively). The findings also showed significantly more bleeding in cases including HCS or combined with RC (*p* = 0.017) (Table 3).

#### 3.2.2. Factors Associated with Early Bleeding

Among the cases in the follow-up group, the median bleeding period was approximately 105 days for the 21 bleeding cases, which were divided into two groups: 11 cases with “early bleeding occurring within 105 days” and 10 cases with “late bleeding occurring after the 105th day.” The factors associated with early bleeding were examined in the same way as the analysis of bleeding-associated factors (Table 4, Table 5, Table 6 and Table 7).

In terms of patient characteristics (Table 4), hepatic functional reserve (Table 5), and background liver (Table 6), no significant difference was found between the early bleeding cases and late bleeding cases. Patient characteristics, hepatic functional reserve, and background liver were not related to the early bleeding factor. Endoscopic findings (Table 7) showed no difference in RC between the two groups. The results in Table 7 show that CRS were less common in early bleeding cases (*p* = 0.67). Only RWM was not related to the early bleeding factor (*p* = 0.012). Furthermore, early bleeding was significantly more common among cases including HCS or combined with RC (*p* = 0.024).

## 4. Discussion

Esophageal varices are a pathological condition resulting from the development of collateral circulation associated with portal hypertension, by which bleeding occurs in 5% to 15% of cases per year [6,8]. Esophageal variceal bleeding can be fatal. Consequently, prediction and preventive treatment are of great importance. Treatment methods for varices include endoscopic treatment, surgical treatment, interventional radiology (IVR), and drug therapy, especially with the administration of NSBBs. In Japan, endoscopic treatment is regarded as the primary method of treatment. Endoscopic treatment consists of EIS and EVL, with the decision based on hemodynamics and the timing of treatment, such as during bleeding, during the waiting period, or for prevention [9]. However, F1 varices with low height are difficult to treat endoscopically, with incomplete treatment leading to recurrence. By contrast, for RC-positive varices, a high risk of bleeding has been demonstrated. Therefore, treatment intervention is conducted at an early stage [10]. No reported study has examined the natural history of F1 and RC-positive varices without treatment of NSBBs. For that reason, the acceptable waiting period duration remains unclear. Our study of follow-up findings from wait-and-see approaches adopted for F1 varices with RC-positivity indicated a bleeding rate as high as 78% and a median time for bleeding as short as 105 days. When treatment intervention was conducted at the time of diagnosis, the bleeding rate was suppressed to as low as 10%. Moreover, the period of sustained absence of bleeding reached a median value of 1156 days, indicating that maintenance of a non-bleeding period was achieved. Our study showed that, even in those cases of F1varices with RC-positive, the time to rebleeding and the period of sustained absence of bleeding can be prolonged by carrying out treatment intervention at an early stage. Therefore, when the RC sign is positive, adequate prophylactic treatment must be conducted early.

Esophageal varices are described based on six items: the occupied area (site), morphology (form), color tone, RC sign, bleeding, and mucosal findings. In particular, RC-positive varices are reportedly at a higher risk of bleeding. An analysis of the association between RC and bleeding showed that, among bleeding cases, RC-positive cases accounted for 93.2%, whereas they accounted for 62.2% among non-bleeding cases. Furthermore, bleeding has reportedly been found in 58.7% of RC-positive cases and in 9.1% of RC-negative cases [11]. For RC classified as CRS, RWM, and HCS, the respective bleeding rates were 59.0%, 73.0%, and 100%, indicating that HCS had the highest bleeding rate [12]. In our study, patients with CRS were few among the bleeding cases in the follow-up group, but cases that included HCS or those with combined forms of RC showed significantly greater bleeding, and they bled at an early stage. These findings suggest that HCS was especially susceptible to bleeding among the types of RC. Although it is reported that enlarged tortuous varices with RC sign or the largest varices with RC sign have a risk of bleeding [11], the paper did not discuss the risk of bleeding of RC sign in small/straight varices. In other words, the result of our study “Even if small/straight varices, combined RC sign or HCS are bleeding-related factors and early bleeding factors” was an important finding that has not been reported previously. Moreover, only RWM was not a bleeding-related factor. There was non-involvement in early bleeding. Early intervention might not be necessary, even if RWM only is detected. Although it might not be necessary to administer NSBBs in cases of small varices with only RWM, a prospective study is necessary to clarify these inferences from earlier findings.

Blood vessels presented with images of RC signs, such as RWM, CRS, and HCS, can rupture and bleed easily [13,14]. Histopathologically, RWM and CRS (which are both RC) are markedly dilated and tortuous very small blood vessels of the lamina propria, RWM are distended red blood vessels overlapping on the surface of varices, and CRS are raised small red spots on the surface of varices. They have fundamentally equivalent characteristics. The superficial layers of the very small dilated blood vessels are only covered with one or several layers of stratified squamous epithelium. In contrast, HCS are large reddened spots protruding from the surface of varices. Histological observation shows that the submucosal vein, a varicose vein, is greatly dilated. It appears to protrude to the surface at one end to push away and dislodge the stratified squamous epithelium; HCS can break more easily, leading to severe bleeding [15]. In our study, when the RC sign was positive, especially when findings showed HCS and RC coexisting, a high bleeding rate and early bleeding were observed, the observations of which were supported by pathological findings.

In addition to the RC and the variceal form, factors such as alcohol consumption, hepatic functional reserve, treatment history, gastric mucosal atrophy (*Helicobacter pylori* infection), and pharmacological agents have also been reported as esophageal variceal bleeding risk factors [16,17,18,19]. In our study of the natural history of F1 varices with RC-positivity, risk factors other than endoscopic findings were not involved in bleeding or early bleeding. Furthermore, this study elucidated the natural history of RC-positive and F1 varices under similar conditions of bleeding risk (patient characteristics, hepatic functional reserve, and background liver).

This study has several limitations. First, this was a retrospective study. If effect size is 0.5, αErr is 0.05, and Power is 0.8 (the x-square test using G Power 3.1.9.7 calculation software), our study needed 32 cases as the sample size. However, the cases in the follow-up group were few because treatment intervention was almost always performed whenever the RC was positive. Because our sample size was small and the selection bias cannot be reduced, it is important to be careful in interpreting our results. So, it will be necessary to consider more cases in the future. Second, no confirmation was made of the local hemodynamics of varices by endoscopic ultrasonography (EUS). Third, the cases examined for this study included patients who had treatment. Regarding observation using EUS, blood vessels outside the esophageal wall are known to be involved in variceal recurrence [20,21]. The presence or absence of development of such blood vessels is believed to be involved in the occurrence of bleeding in patients with F1 varices with RC-positivity. Considering the risk of bleeding in esophageal varices during follow-up is extremely important, as is obtaining definitive evidence pertaining to this study. A large-scale prospective study that also examines the local hemodynamics of esophageal varices (inside and outside the esophageal wall) must be conducted.

## 5. Conclusions

In conclusion, when the RC sign is positive, early treatment intervention might be needed, even with F1 varices. If RWM only is recognized, early intervention might not be necessary. However, because HCS in particular is a factor associated with bleeding and early bleeding, the response in such cases might be faster.

## Figures and Tables

**Table 1:** Patient characteristics (*n* = 76).

Age, Sex, Underlying Illnesses, Preferences		Liver Cancer, Liver Function and Hemodynamics	
Age, years, median value	62.0 (43–82)	Child–Pugh A/B/C, *n* (%)	36 (47)/28 (37)/12 (16)
Sex, male, *n* (%)	49 (64)	Mean platelet count (×10^4^/µL)	11.5
Diabetes mellitus, *n* (%)	21 (28)	Hepatocellular carcinoma, *n* (%)	18 (24)
Hypertension, *n* (%)	17 (22)	VP 0/1/2/3/4, *n* (%)	67 (88)/3 (4)/4 (6)/1(1)/1(1)
Hyperlipidemia, *n* (%)	4 (6)	Portal thrombosis, *n* (%)	4 (5)
Antithrombotic therapy/NSAIDs, *n* (%)	13 (17)/10 (13)	Shunt, *n* (%)	32 (42)
Prior administration of PPI, *n* (%)	61 (82)	Variceal inflow vessels: left gastric vein only/posterior gastric vein only/multiple, *n* (%)	57 (75)/6 (8)/13 (17)
Alcohol consumption, *n* (%)	10 (13)		
Gastric mucosal atrophy, *n* (%)	53 (70)		
** Background liver **		** Endoscopic findings **	
Alcohol, *n* (%)	13 (17)	Treatment history, *n* (%)	42 (55)
Hepatitis C, *n* (%)	18 (24)	RC 1/2/3, *n* (%)	50 (66)/22 (29)/4 (5)
Hepatitis B, *n* (%)	9 (12)	RWM, *n* (%)	27 (36)
NASH, *n* (%)	6 (8)	CRS, *n* (%)	24 (32)
PBC, *n* (%)	5 (7)	HCS or combined, *n* (%)	25 (33)
Others, *n* (%)	25 (33)		

Abbreviations: NSAIDs, non-steroidal anti-inflammatory drugs; PPI, proton pump inhibitor; NASH, non-alcoholic steatohepatitis; PBC, primary biliary cholangitis; VP, portal vein invasion; RC, red color sign; RWM, red wale marking; CRS, cherry red spot; HCS, hematocystic spot.

**Table 2:** Patient characteristics: Treatment intervention group (*n* = 49) and follow-up group (*n* = 27).

	Treatment Intervention Group	Follow-up Group	*p*-Value
** Period of observation, ** ** days ** ** (median) **	792 (180–2920)	152 (1–2500)	<0.001
** Age, Sex, underlying illnesses, preferences **			
Age, years, median value	62.0 (45–80)	62.8 (43–82)	0.72
Sex, male, *n* (%)	32 (65)	17 (63)	0.52
Diabetes mellitus, *n* (%)	10 (20)	11 (41)	0.98
Hypertension, *n* (%)	11 (22)	6 (22)	0.61
Hyperlipidemia, *n* (%)	3 (6)	1 (4)	0.55
Antithrombotic therapy/NSAIDs, *n* (%)	6 (12)/6 (12)	7 (26)/4 (18)	0.97/0.75
Prior administration of PPI, *n* (%)	41 (83)	20 (74)	0.24
Alcohol consumption, *n* (%)	3 (6)	7 (26)	0.10
Gastric mucosal atrophy, *n* (%)	32 (65)	21 (78)	0.92
** Background liver **			
Alcohol, *n* (%)	8 (16)	5 (19)	0.72
Hepatitis C, *n* (%)	12 (24)	6 (22)	0.53
Hepatitis B, *n* (%)	6 (12)	3 (11)	0.60
NASH, *n* (%)	3 (6)	3 (11)	0.89
PBC, *n* (%)	2 (4)	3 (11)	0.95
Others, *n* (%)	18 (37)	7 (26)	0.24
** Liver cancer, liver function and hemodynamics **			
Child–Pugh A/B/C, *n* (%)	27 (55)/17 (35)/5 (10)	9 (33)/11 (41)/7 (26)	0.06/0.78/0.98
Mean platelet count (×10^4^/µL)	11.2	11.7	0.90
Hepatocellular carcinoma, *n* (%)	9 (18)	9 (33)	0.96
VP 0/1/2/3/4, *n* (%)	47 (96)/2 (4)/0 (0)/0 (0)/0 (0)	20 (74)/1 (4)/4 (14)/1 (4)/1 (4)	0.008/0.71/1.00/1.00/1.00
Portal thrombosis, *n* (%)	3 (6)	1 (4)	0.55
Shunt, *n* (%)	18 (37)	14 (52)	0.94
Variceal inflow vessels: left gastric vein only/posterior gastric vein only/multiple, *n* (%)	35 (71)/3 (6)/11 (23)	22 (81)/3 (11)/2 (7)	0.90/0.89/0.085
** Endoscopic findings **			
Treatment history, *n* (%)	25 (51)	17 (63)	0.89
RC 1/2/3, *n* (%)	30 (61)/17 (35)/2 (4)	20 (74)/5 (19)/2 (7)	0.92/0.11/0.88
RWM, *n* (%)	20 (41)	7 (26)	0.15
CRS, *n* (%)	16 (33)	8 (30)	0.50
HCS or combined, *n* (%)	13 (26)	12 (44)	0.97
**Bleeding rate and median period of sustained absence of bleeding**			
Bleeding rate, *n* (%)	5 (10)	21 (78)	<0.001
Median period of sustained absence of bleeding, days (median)	1156 (274–1582)	105 (1–336)	<0.001

Abbreviations: NSAIDs, non-steroidal anti-inflammatory drugs; PPI, proton pump inhibitor; NASH, non-alcoholic steatohepatitis; PBC, primary biliary cholangitis; VP, portal vein invasion; RC, red color sign; RWM, red wale marking; CRS, cherry red spot; HCS, hematocystic spot.

**Table 3:** Bleeding-associated factors in the follow-up group: Endoscopic findings.

	Overall *n* = 27	Bleeding Group *n* = 21	Non-Bleeding Group *n* = 6	*p*-Value	Cramer V
Treatment history, *n* (%)	17 (63)	11 (52)	6 (100)	0.057	-
RC 1/2/3, *n* (%)	20 (74)/5 (19)/2 (7)	16 (73)/3 (14)/2 (9)	4 (67)/2 (33)/0 (0)	0.50/0.30/0.60	-
RWM, *n* (%)	7 (26)	5 (24)	2 (33)	0.50	-
CRS, *n* (%)	8 (30)	4 (19)	4 (67)	0.04	0.43
HCS or combined, *n* (%)	12 (44)	12 (57)	0 (0)	0.017	0.48

Abbreviations: RC, red color sign; RWM, red wale marking; CRS, cherry red spot; HCS, hematocystic spot.

**Table 4:** Factors associated with early bleeding in the follow-up group.

	Overall *n* = 21	Early Group *n* = 11	Late Group *n* = 10	*p*-Value
Age, years, median value	61.9 (43–82)	63 (49–75)	61 (43–82)	0.43
Sex, male, *n* (%)	14 (67)	7 (64)	7 (70)	0.56
Diabetes mellitus, *n* (%)	10 (48)	6 (55)	4 (40)	0.41
Hypertension, *n* (%)	4 (19)	3 (27)	2 (20)	0.55
Hyperlipidemia, *n* (%)	1 (5)	1 (9)	0(0)	0.52
Antithrombotic therapy/NSAIDs, *n* (%)	6 (29)/3 (14)	4 (36)/1 (19)	2 (20)/2 (20)	0.37/0.46
Prior administration of PPI, *n* (%)	15 (71)	8 (73)	7 (70)	0.63
Alcohol consumption, *n* (%)	6 (29)	3 (27)	3 (30)	0.63
Gastric mucosal atrophy, *n* (%)	16 (76)	9 (82)	7 (70)	0.45

Abbreviations: NSAIDs, non-steroidal anti-Inflammatory drugs; PPI, proton pump inhibitor.

**Table 5:** Factors associated with early bleeding in the follow-up group: liver cancer, hepatic functional reserve and hemodynamics.

	Overall *n* = 21	Early Group *n* = 11	Late Group *n* = 10	*p*-Value
Child–Pugh A/B/C, *n* (%)	5 (24)/9 (43)/7 (33)	2 (18)/4 (37)/5 (45)	3 (30)/5 (50)/2 (20)	0.45/0.43/0.22
Mean platelet count (×10^4^/µL)	11.9	12.3	11.6	0.66
Hepatocellular carcinoma, *n* (%)	8 (38)	2 (8)	6 (60)	0.063
VP 0/1/2/ 3/4, *n* (%)	15 (71)/1 (5)/3 (14)/ 1 (5)/1 (5)	9 (82)/0 (0)/0 (0)/ 1 (9)/1 (9)	6 (60)/1 (10)/3 (30)/ 0 (0)/0 (0)	0.27/0.48/0.090/ 0.52/0.52
Portal thrombosis, *n* (%)	1 (5)	0 (0)	1 (10)	0.48
Shunt, *n* (%)	11 (52)	5 (45)	6 (60)	0.41
Variceal inflow vessels: left gastric vein only/posterior gastric vein only/multiple, *n* (%)	17 (80)/2 (10)/2 (10)	9 (82)/1 (9)/1 (9)	8 (80)/1 (10)/1 (10)	0.67/0.74/0.74

Abbreviations: VP, portal vein invasion.

**Table 6:** Factors associated with early bleeding in the follow-up group: background liver.

	Overall *n* = 21	Early Group *n* = 11	Late Group *n* = 10	*p*-Value
Alcohol, *n* (%)	5 (24)	2 (18)	3 (30)	0.45
Hepatitis C, *n* (%)	5 (24)	2 (18)	3 (30)	0.45
Hepatitis B, *n* (%)	2 (10)	1 (9)	1 (10)	0.74
NASH, *n* (%)	3 (14)	1 (9)	2 (20)	0.46
PBC, *n* (%)	3 (14)	3 (27)	0 (0)	0.12
Others, *n* (%)	3 (14)	2 (18)	1 (10)	0.54

Abbreviations: NASH, non-alcoholic steatohepatitis; PBC, primary biliary cholangitis.

**Table 7:** Factors associated with early bleeding in the follow-up group: endoscopic findings.

	Overall *n* = 21	Early Group *n* = 11	Late Group *n* = 10	*p*-Value	Cramer V
Treatment history, *n* (%)	11 (52)	6 (55)	5 (50)	0.59	-
RC 1/2/3, *n* (%)	16 (73)/3 (14)/2 (9)	7 (63)/3 (27)/1 (9)	9 (90)/0 (0)/1 (1)	0.19/0.12/0.74	-
RWM, *n* (%)	5 (24)	0 (0)	5 (50)	0.012	0.59
CRS, *n* (%)	4 (19)	2 (18)	2 (20)	0.67	-
HCS or combined, n (%)	12 (57)	9 (81)	3 (30)	0.024	0.52

Abbreviations: RC, red color sign; RWM, red wale marking; CRS, cherry red spot; HCS, hematocystic spot.

## Data Availability

Not applicable.

## Supplementary Materials

The following supporting information can be downloaded at: https://www.mdpi.com/article/10.3390/healthcare10071193/s1, Table S1: Bleeding-associated factors in the follow-up group patient characteristics; Table S2: Bleeding-associated factors in the follow-up group: Liver cancer, hepatic functional reserve and hemodynamics; Table S3: Bleeding-associated factors in the follow-up group: Background liver.
