# Supplementary material for: The Risk of Bleeding in Small/Straight Esophageal Varices with Red Color Sign on Endoscopy: A Retrospective Analysis from the Natural Course
Source: Healthcare (Basel). 2022 Jun 26;10(7):1193. doi: 10.3390/healthcare10071193 (PMC9322794; doi:10.3390/healthcare10071193)
Supplement: Supplementary file 1 [file healthcare-10-01193-s001.zip › healthcare-1740751-supplementary.pdf]

Supplementary Table S1. Bleeding-associated factors in the follow-up group patient characteristics

	**Overall** *n* = 27	**Bleeding group** *n* = 21	**Non-bleeding group** *n* = 6	***P*-value**
**Period of observation, days (median)**	152	105 (1–336)	1930 (365–2500)	< .001
Age, years (median value)	62.8 (43–82)	61.9 (43–82)	66 (54–80)	.76
Sex, *n* (male, %)	17 (63)	14 (29)	3 (11)	.39
Diabetes mellitus, *n* (%)	11 (41)	10 (48)	1 (17)	.19
Hypertension, *n* (%)	6 (22)	4 (19)	2 (33)	.40
Hyperlipidemia, *n* (%)	1 (4)	1 (5)	0 (0)	.78
Antithrombotic therapy / NSAIDs, *n* (%)	7 (26) / 4 (18)	6 (29) / 3 (14)	1 (17) / 1 (17)	.50 / .66
Prior administration of PPI, *n* (%)	20 (74)	15 (71)	5 (83)	.50
Alcohol consumption, *n* (%)	7 (26)	6 (29)	1 (27)	.50
Gastric mucosal atrophy, *n* (%)	21 (78)	16 (76)	5 (83)	.60

Abbreviations: NSAIDs, non-steroidal anti-inflammatory drugs; PPI, proton pump inhibitor.

Supplementary Table S2. Bleeding-associated factors in the follow-up group: Liver cancer, hepatic functional reserve and hemodynamics

	**Overall** *n* = 27	**Bleeding group** *n* = 21	**Non-bleeding group** *n* = 6	***P*-value**
Child–Pugh A /B / C, *n* (%)	9 (33) / 11 (41) / 7 (26)	5 (23) / 9 (41) / 7 (32)	4 (67) / 1 (17) / 0 (0)	.073 / .55 / .13
Mean platelet count (×10^4/μL)	11.7	11.9	11.4	.80
Hepatocellular carcinoma, *n* (%)	9 (33)	8 (38)	1 (17)	.32
VP 0 /1 / 2/ 3 / 4, *n* (%)	20 (74) / 1 (4) / 4 (14) / 1 (4) / 1 (4)	15 (71) / 1 (5) / 3 (14) / 1 (5) / 1 (5)	5 (83) / 0 (0) / 1 (17) / 0 (0) / 0 (0)	.50 / .78 / .66 / .78 / .78
Portal thrombosis, *n* (%)	1 (4)	1 (5)	0 (0)	.78
Shunt, *n* (%)	14 (52)	11 (52)	3 (50)	.50
Variceal inflow vessels: left gastric vein only / posterior gastric vein only / multiple, *n* (%)	22 (81) / 3 (11) / 2 (7)	17 (77) / 2 (9) / 2 (9)	5 (83) / 1 (17) / 0 (0)	.50

Abbreviations: VP, portal vein invasion.

Supplementary Table S3. Bleeding-associated factors in the follow-up group: Background liver

	**Overall** *n* = 27	**Bleeding group** *n* = 21	**Non-bleeding group** *n* = 6	***P*-value**
Alcohol, *n* (%)	5 (19)	5 (24)	0 (0)	.25
Hepatitis C, *n* (%)	6 (22)	5 (24)	1 (17)	.60
Hepatitis B, *n* (%)	3 (11)	2 (10)	1 (17)	.55
NASH, *n* (%)	3 (11)	3 (14)	0 (0)	.46
PBC, *n* (%)	3 (11)	3 (14)	0 (0)	.46
Others, *n* (%)	7 (26)	3 (14)	4 (66)	NA

Abbreviations: NASH, non-alcoholic steatohepatitis; PBC, primary biliary cholangitis; NA, not applicable
